# Widely-tunable synchronisation-free picosecond laser source for multimodal CARS, SHG, and two-photon microscopy

**DOI:** 10.1364/BOE.411620

**Published:** 2021-01-26

**Authors:** Duanyang Xu, Sijing Liang, Lin Xu, Konstantinos N. Bourdakos, Peter Johnson, James Read, Jonathan H. V. Price, Sumeet Mahajan, David J. Richardson

**Affiliations:** 1 Optoelectronics Research Centre, University of Southampton, Southampton, SO17 1BJ, UK; 2 Institute for Life Sciences, University of Southampton, Southampton, SO17 1BJ, UK; 3 Institute for Life Sciences and School of Chemistry, University of Southampton, Southampton, SO17 1BJ, UK

## Abstract

We demonstrate a continuous wave (CW) seeded synchronization-free optical parametric amplifier (OPA) pumped by a picosecond, 1 µm laser and show its performance when used as a simple yet powerful source for label-free coherent anti-Stokes Raman scattering (CARS), concurrent second harmonic generation (SHG), and two-photon fluorescence microscopy in an epi-detection geometry. The average power level of above 175 mW, spectral resolution of 8 cm^−1^, and 2 ps pulse duration are well optimized for CARS microscopy in bio-science and bio-medical imaging systems. Our OPA is a much simpler setup than either the “gold-standard” laser and optical parametric oscillator (OPO) combination traditionally used for CARS imaging, or the more recently developed OPA systems pumped with femtosecond pulses [1]. Rapid and accurate tuning between resonances was achieved by changing the poled channels and temperature of the periodically-poled lithium niobate (PPLN) OPA crystal together with the OPA seed wavelength. The Pump-Stokes frequency detuning range fully covered the C-H stretching band used for the imaging of lipids. By enabling three multiphoton techniques using a compact, synchronization free laser source, our work paves the way for the translation of label-free multi-photon microscopy imaging from biomedical research to an imaging based diagnostic tool for use in the healthcare arena.

## 1. Introduction

Laser-based spectroscopy and imaging has been widely used, becoming an almost essential tool in biochemical and medical applications. Despite the many attractive features of the spectroscopically specific CARS imaging modality, its uptake is restricted by the cost, size and complexity of the ‘gold-standard’ synchronously pumped OPO systems currently used [2]. To address the need for sources that output the required few-ps pulse widths considered optimal for CARS bioimaging [3], progress has been achieved using spectral-focusing CARS [4] based on either supercontinuum [5] or a single < 20 fs laser with sufficient bandwidth [6], but those solutions still have present technical obstacles, so they have not achieved wide acceptance.

CARS is label-free and has significant advantages including chemically specificity and z-sectioning capability compared to the prevalent fluorescence based one-photon imaging methods. It also offers significantly faster signal acquisition times compared to Raman spectroscopy [3,7]. In the field of biological imaging, it is an almost ideal method for studying lipids and their metabolism [8], and it is able to distinguish lipids from proteins via its contrast mechanism [9]. CARS can also be combined with other nonlinear imaging methods such as two-photon excited fluorescence microscopy (TPEF) [10] and second harmonic generation (SHG) [11] on a single microscope system using a multi-channel output scheme [12–14]. Combining these imaging methods has been done via a technique called multimodal-CARS, which has proved to be beneficial in a variety of applications that require structure- and chemical-specific imaging contrast because each mode can isolate various sample details [15–18] and image live cells and tissue [19].

CARS is a nonlinear optical process and requires pulsed lasers at a pump frequency and at an offset Raman-Stokes frequency. The anti-Stokes signal is generated when the pump and Stokes are offset by the Raman resonance frequency of the molecule of interest. In order to efficiently drive the vibrational coherence and thus to achieve rapid image acquisition, CARS requires incident pulses of pump and Stokes be temporally and spatially overlapped when tightly focused into the sample. In general, pulses of a few ps duration are optimal for CARS bio-imaging because they have bandwidths that match well with the Raman resonance peak in typical cellular structures and yet still provide the high peak powers needed to efficiently excite this nonlinear process. Due to the technical complexity, high cost and large footprint of such bulk laser based OPO sources, a dedicated optics laboratory is normally required for CARS microscopy, which prevents it from being more widely used. There is an urgent need for a simplified laser system that will provide high performance as needed for CARS but in a small and robust form while at low cost.

A notable recent development was the report of a femtosecond crystal-based laser pump and a CW-seeded PPLN-based OPA that provided high gain and was even sufficiently stable for SRS microscopy without a balanced detection system [1]. The 1520-1640 nm OPA output was frequency doubled using spectral compression (which is lossy) in a PPLN SHG stage to provide the ps pump beam. It also required a narrow spectral filter on the residual beam from the fs pump to broaden its temporal width to the few ps range, which added complexity and reduced the efficiency.

In our work, we incorporate several aspects of that crystal-based OPA system design [1], but simplify the setup further by using a 2-ps OPA pump laser to enable us to both avoid wasting power and to use commercial, off-the-shelf crystals for both the OPA and SHG stages. The CW source is tunable across the entire Raman lipid band in biological tissues (∼ 2700 - 3200 ${\mathrm{cm}}^{-1}$). Tuning is mainly limited by the poling periods of the nonlinear crystals available to us and it could easily be broadened to enable tuning across the protein resonance band (∼ 1430 - 1550 ${\mathrm{cm}}^{-1}$) by specifying a custom crystal with the required poling patterns to improve the system in the future.

We found that in addition to providing good CARS imaging contrast, the OPA also enabled concurrent SHG and TPEF imaging. The powers and beam quality were both well optimized, enabling us to obtain strong signals in the backward direction compared to the signal beam (epi-detection) rather than using forward detection, which is easier due to the strong forward signal, but requires thin samples to allow the signal to be collected in that direction. Hence the system is suitable for imaging clinical-diagnostics-relevant and simple-to-prepare thick samples. The system was tested initially on chemically pure beads to verify the chemically specificity enabled by CARS-spectroscopy. Then, by imaging mouse adipose tissue with epi-detection, the multimodal capabilities were shown in bio-images combining concurrent CARS, SHG and TPEF signals detected in 3 separate output channels on our inverted microscope platform.

## 2. Materials and methods

**Fig. 1.**
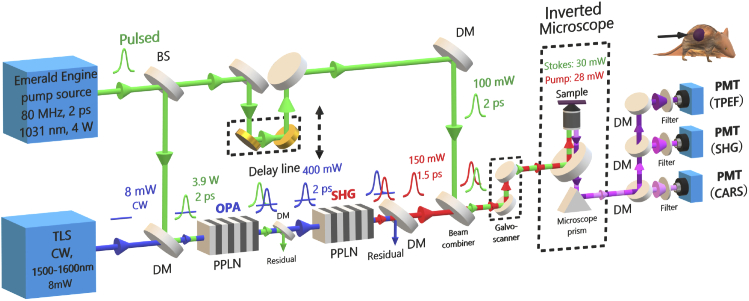
Schematic of the experimental system used for multimodal CARS. BS, beam splitter; DM, dichroic mirror; PPLN, periodically-poled lithium niobate; PMT, photomultiplier tube.

**Fig. 2.**
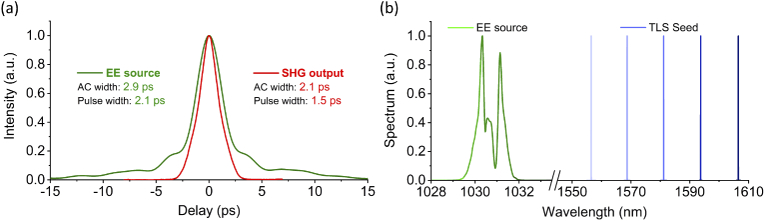
(a) Auto-correlation measurement of EE source (Emerald Engine pump laser) (1031 nm) (green) and OPA-SHG output (red). AC, auto-correlation. (b) Spectrum of the Stokes beam (OPA pump source) and TLS output (OPA seed). (See Fig. 4 for the OPA spectrum.)

**Fig. 3.**
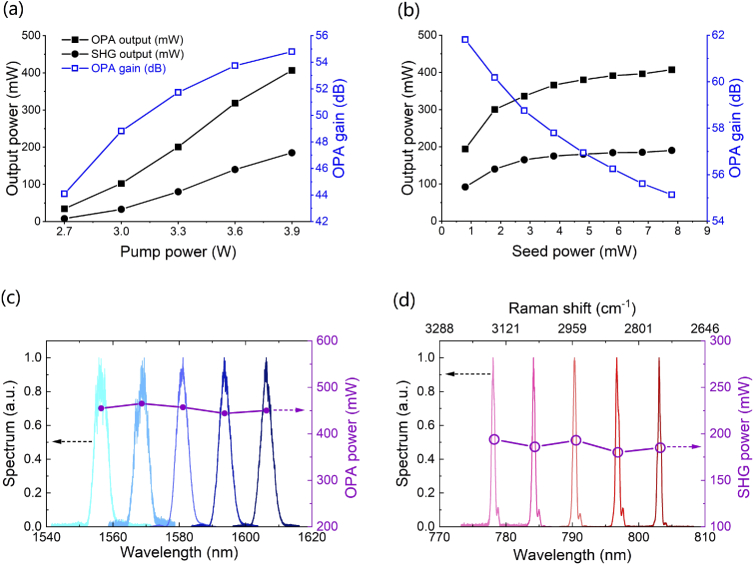
OPA output (solid square), SHG output (solid circle) and OPA gain (empty square, blue) as a function of pump power (a) or seed power (b). Spectra and power of CW seeded OPA (c) and SHG (d) later used as the tunable CARS Pump beam.

The multimodal CARS laser source and microscope system is shown in Fig. 1. The pump laser was an APE Emerald Engine laser, the output of which (∼ 4 W, 2 ps, 80 MHz, 1031 nm) was split into two beams: one for pumping the OPA and another for the Stokes beam used in the CARS imaging work. The OPA was seeded by a semiconductor-based CW tunable laser (TLS: Photonetics Osics 3610 RA00) with fiberized output, which generated <10 mW average power in the 1500-1600 nm range. The OPA pump and seed were collimated and collinearly focused to a 75-µm waist in a 20-mm-long MgO-doped periodically poled lithium niobate (PPLN) crystal with 5 different poling periods from Λ=29.52 µm to Λ=31.59 µm in 0.5 µm steps (Covesion: MOPO1-1.0-20). Adjusting the wavelength of the TLS then selecting the correct poling period and temperature for the PPLN crystal enabled quick and precise tuning of the OPA. An output power above 400 mW across the entire OPA tuning range was achieved (Fig. 3(c)). The spectral linewidth of the OPA output varied from 17 ${\mathrm{cm}}^{-1}$ at 1556 nm to 13 ${\mathrm{cm}}^{-1}$ at 1606 nm, approximately commensurate with the pulse duration carved by the pump. The OPA output pulses had a measured duration of 2.1 ps (data not shown in Fig. 2), which is similar to that of the pump. There were approximately 1.2 × ${\mathrm{10}}^{5}$ photons from the CW seed laser within the time window of each pump pulse, which is sufficient to completely suppress the noisier parametric superfluorescence generation process in the OPA [20]. Pumping with a low noise solid state laser and using the CW seed thus means the OPA will have both minimal spectral drift vs. time and low temporal jitter.

The output signal of the OPA was separated from the residual pump and was focused into a second PPLN crystal for SHG, which provides the pump beam for CARS imaging. The SHG PPLN crystal had a length of 10 mm and 5 grating periods from Λ=19.2 µm to Λ=20.4 µm in step of 0.3 µm)(Covesion: MSHG1550-1.0-10). The tuning range of the SHG output was 775 - 805 nm, limited by the SHG crystal available and thus can be extended to cover the full OPA tuning range by selecting appropriate poling periods in the future. The SHG pulse duration (operating wavelength of 1594.4 nm) was ∼1.5 ps (as shown in Fig. 2(a)). The pump-Stokes frequency detuning range from 2700 to 3200 ${\mathrm{cm}}^{-1}$ fully covered the C-H stretching band that is typically used for the imaging of lipids. A delay line in the pump beam path and dichroic mirrors (DMs) were used to combine and overlap the Pump and Stokes pulses temporally and spatially; both the beams were delivered collinearly into a home-built CARS microscope system. The inverted microscope was operated in reflection (epi) mode using an air objective lens with numerical aperture (NA) of 0.75 (Nikon 20×) to focus the Pump and Stokes on the sample. A galvo scanner was employed for fast spatial scanning of the beam. The epi light was collected and detected (in non-descanned fashion) by three photo-multiplier tubes (filters: 400±20 nm for SHG, 520±20 nm for TPEF, and 640±20 nm for CARS).

## 3. Results and discussion

### 3.1 OPA development

We first tested the OPA performance as a function of pump power and seed power. When the seed laser was set with wavelength of 1594.4 nm and output power of 8 mW, the minimum pump needed for gain to be measured was 2.7 W and the OPA output power increased with pump power beyond this reaching 400 mW at the maximum pump power of 3.9 W, as shown in Fig. 3(a). (The maximum pump power was set to stay below the damage threshold of the PPLN crystal.) The corresponding gain of the OPA increased with pump power to a maximum of 55 dB. We also characterized the performance of the gain with respect to the seed power in the OPA when the pump was fixed at 3.9 W. As shown in Fig. 3(b), the output power increased and the gain decreased vs. increasing seed power with saturation observed at seed powers above ∼5 mW. Figure 3 also shows the SHG output power characteristics. The maximum SHG output power was 185 mW. The tunable output in the 1540 - 1620 nm range is shown in Fig. 3(c). Output powers of close to 450 mW were achieved across the whole tuning range. The OPA spectrum had a FWHM bandwidth of 4 nm, which results in a time-bandwidth product of 0.98 (∼3 times the transform-limited value).

Figure 3(d) shows the SHG spectra spanning from 775 nm to 805 nm. The output power is more than sufficient for the CARS, SHG and TPEF bio-imaging considering the power limitations imposed by photo bio-toxicity. As shown in Fig. 3(d) the average power is in the 175 - 200 mW range throughout the tuning range. Due to the 1 nm pump spectral acceptance bandwidth of the SHG crystal being smaller than the pump spectral bandwidth, the SHG process cleaned up the chirp of the pump and reduced the width of the pump spectra and pulse. The SH spectrum had a FWHM bandwidth of 0.5 nm (8 ${\mathrm{cm}}^{-1}$) and the measured SHG pulse duration was 1.5 ps (time-bandwidth product = 0.35, which is improved to ∼1.11 times of the transform-limited value). The output beam was close to a Gaussian profile with $M_{x}^{2}$ measured to be 1.29 and $M_{y}^{2}$ = 1.18.

We used the tunable laser source (TLS) to demonstrate the wavelength flexibility of the OPA. Note that we also did experiments with a much less costly 1592 nm wavelength telecoms laser diode with similar power to the TLS and temperature-tuned the diode wavelength by up to 6 nm and achieved similar output power and pulse characteristics from the OPA as when using the TLS. (These telecoms diodes are readily available commercially at a very small fraction of the cost of a TLS and are much more compact.) This verified that, in principle, the costs of the system could be reduced and the tuning range maintained by using a switchable array of telecoms seed diodes with the wavelengths selected to target different molecular vibrational frequencies in the bio-samples of interest.

### 3.2 Chemical imaging results

**Fig. 4.**
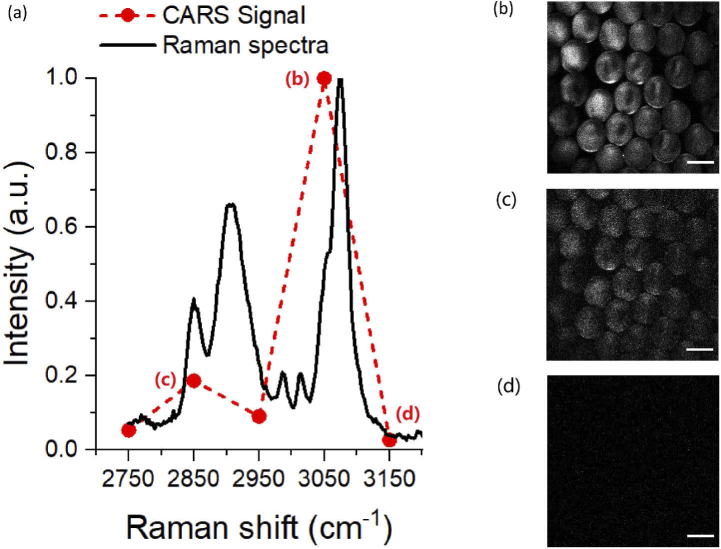
(a) Measured CARS spectra of polystyrene beads overlayed with the spontaneous Raman spectra. (b) (c) (d) show the corresponding polystyrene CARS images vs Raman excitation frequency 3050 ${\mathrm{cm}}^{-1}$, 2850 ${\mathrm{cm}}^{-1}$ and 3150 ${\mathrm{cm}}^{-1}$ respectively (scale bar: 40 µm).

We next tested the chemical specificity and imaging ability of our system by recording the CARS spectra and microscopy images of 40-µm diameter polystyrene beads. The SHG of the OPA output was tuned from 778 to 803 nm enabling the CARS anti-Stokes signals shown in Fig. 4 to be probed at frequencies from 2750 to 3150 ${\mathrm{cm}}^{-1}$. The general profile of the CARS spectrum and the position of the two characteristic peaks at 2850 ${\mathrm{cm}}^{-1}$ and 3050 ${\mathrm{cm}}^{-1}$ are in reasonable agreement with the spontaneous Raman spectrum. (CARS peaks are a convolution of the signal with the non-resonant background with a dispersive shape, so the peaks are not expected to be coincident with spontaneous Raman.)

The spontaneous Raman reference spectrum for polystyrene was recorded with a home-built Raman micro-spectrometer system utilizing a Shamrock303 spectrograph with an Andor iDus 420 camera as the detector and a 785 nm excitation laser. The spectrum was acquired with a single 10 second exposure (50 µm slit, ∼4 ${\mathrm{cm}}^{-1}$ resolution) with 15 mW laser power using a 40× (0.9 NA) objective. The average power levels on the sample were 6 mW (Pump) and 12 mW (Stokes). The pixel dwell time was 8 µs, so that the acquisition of a full image (512 × 512 pixels) took ∼ two seconds. Good image contrast is seen at the strongest resonance frequency in Fig. 4(b) as compared to the weaker resonance in Fig. 4(c) and the off resonance data in Fig. 4(d).

### 3.3 Biological imaging results

We next demonstrate bio-imaging by showing CARS along with SHG and two-photon autofluorescence (TPEF) imaging in an epi-detection configuration. Biological samples were prepared by excising tissues from culled mice. All procedures were carried out in accordance with the Animals (Scientific Procedures) Act 1986 set out by the UK Home Office. Female C57BL/6 mice aged between 4-6 months were culled via ${\mathrm{CO}}_{2}$ and cervical dislocation. The perigonadal adipose tissue was removed using scissors and a pair of forceps. The tissue was fixed in 4% paraformaldehyde solution for 4 hours then washed in phosphate-buffered saline (PBS) immediately before being placed in a glass bottomed dish with a coverslip (#1.5; 0.17 mm thick) imaging window.

**Fig. 5.**
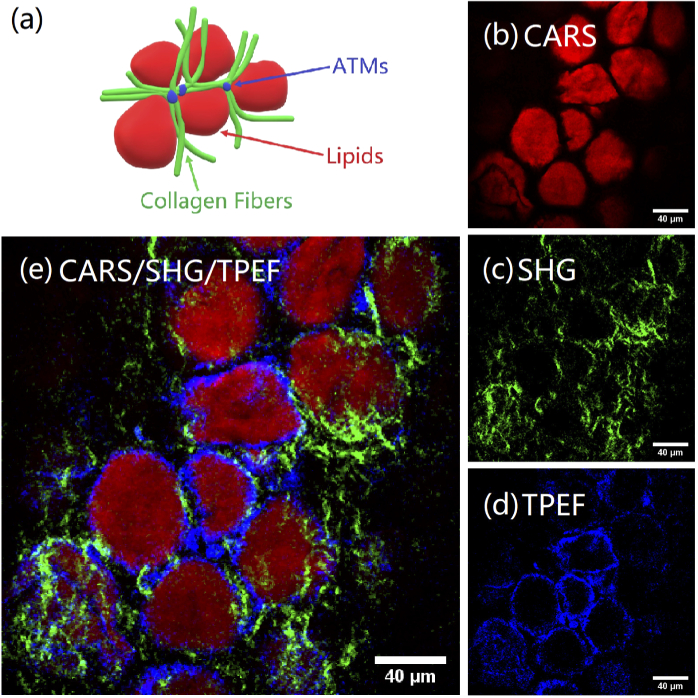
Multimodal CARS/SHF/TPEF bioimaging with our new OPA/SHG source. Images are of mouse adipose tissue acquired at the surface of the sample. (a) Schematic showing the main components of adipose tissue; (b) CARS image mapping the intensity of the 2845 ${\mathrm{cm}}^{-1}$ lipid band across the tissue; (c) collagen fibers imaged with SHG; (d) TPEF images FAD autofluorescence in macrophage cells; (e) composite image with CARS, SHG and TPEF overlaid. Color intensity relates linearly to the counts on the detector, normalized to the maximum to obtain the highest contrast for each of the channels independently. The images shown are single frame CARS acquisitions with pixel dwell time of 8 µs, 28 mW pump and 30 mW of Stokes.

Representative images for adipose tissue from mice are shown in Fig. 5. Image acquisition was carried out such that CARS and SHG and TPEF images were simultaneously acquired on our three-channel detection setup. Each modality provides a different chemical or structural readout. It should be noted that all three signals are from endogenous contrast mechanisms inherent to the sample, and no dyes or stains were added to enhance contrast.

Images are acquired with a Pump-Stokes frequency detuning of 2845 ${\mathrm{cm}}^{-1}$ (Pump: 797.2 nm, Stokes: 1031 nm). The average power levels on the sample are 28 mW Pump and 30 mW Stokes. The pixel dwell time is 8 µs, so that the acquisition of a full image (512 × 512 pixels) takes ∼ two seconds. A schematic of the expected arrangement of different components in adipose tissue is shown in Fig. 5(a). It can be seen from the CARS channel (Fig. 5(b)) that the signal from the ${\mathrm{CH}}_{2}$ peak at 2845 ${\mathrm{cm}}^{-1}$ is diffuse and not structurally dependent, as it is from the uniform lipid distribution within the adipocyte (fat cell). Indeed, lipids are rich in C-H bonds resonant at this frequency so CARS microscopy allows selective imaging of lipid droplets in unstained live cells with very high contrast. Figure 5(c) shows the corresponding SHG image. SHG interrogates the $\chi^{\left(2\right)}$-related part of the hyperpolarizability (second-order electric susceptibility per unit volume) of materials. In this image the SHG was generated by the Pump as verified by turning off the Stokes beam and confirming the SHG channel signal was still there. SHG from only Stokes or SFG from Pump and Stokes would potentially be observable using a different choice of filters for the respective channel. Here, SHG selectively images collagen fibers, which are a dominant component of the adipose tissue scaffolding [21], (detection in the range of 400±20 nm) highlighting the fibrillary nature and periodicity of collagen fibers in the sample. We used TPEF to image auto fluorophores (detection in the range of 520±20 nm) in the tissue (flavin adenine dinucleotide (FAD) and flavin mononucleotide (FMN) [22]). These auto fluorophores indicate metabolic activity in cells. The TPEF images in Fig. 5(d) show adipose tissue macrophages (ATMs) that are localized in between adipocyte cells. Blood vessels would also have good contrast with this modality and form a network around adipocytes. Elastin fibers are key components of blood vessel architecture and exhibit TPEF in the spectral range imaged here [23]. We verified that the TPEF signal was neither delay dependent nor did it reduce when the Stokes was blocked so it is not a four-wave mixing signal. The shape and distribution of the macrophages are similar to observations by others [22] and our detection window covers the expected emission spectral range [24]. Figure 5(e) show the composite multimodal image of the same area of the tissue. The multimodal image thus gives the underlying chemical and structural distribution and the local adipose tissue architecture [22] through combined CARS, SHG and TPEF modalities.

**Fig. 6.**
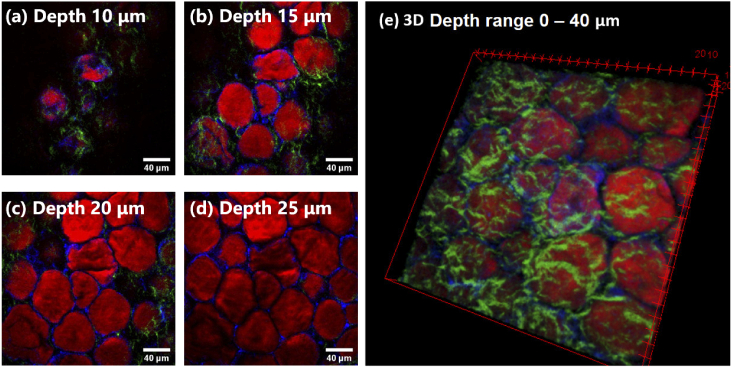
Multimodal imaging at different depths. Composite images with the combined modalities of CARS (red), SHG (green) and TPAF (blue) corresponding to the image shown in Fig. 5 are shown at different depths (a) 10 µm, (b) 15 µm, (c) 20 µm and (d) 25 µm. (e) is the 3D construction of the image (1 µm spacing between each section for 40 slices).

Currently tissue diagnostics makes use of colored stains on thin tissue slices (typically 5-7 µm in thickness) which are suited for the transmission images recorded so that samples require very careful preparation and sectioning. The ability to use label-free imaging on clinically relevant thick tissue samples for diagnostic applications (for example, fresh from surgery) would be greatly simplified if the epi-detection became viable. To highlight this capability, we show in Fig. 6 that our OPA-pumped multimodal CARS imaging setup can image at depths of up to 40 µm (thick tissue samples) without compromising on contrast or localized depth sectioning capability, which is beneficial as surface-sectioning can miss crucial information.

The axial depth of focus is expected to be different for CARS (three-photon process) and SHG/TPEF (two-photon processes): However, with the 1 µm z-resolution of our microscope stage we did not observe any significant effect. Overall, the ability to concurrently generate high contrast images with multiple modalities using the new OPA based laser source should make it a valuable tool for bio-science users. The epi-detection capability with an OPA-based setup demonstrates the relevance to biological and clinical samples without the need for extensive sample preparation which would be required to create the thin sections normally used with forward detection.

## 4. Summary

In summary, we show the demonstration of a compact and reliable, tunable, CW seeded synchronization-free PPLN OPA with a robust, commercial ps pump laser. This laser and OPA combination is shown to be well suited for label-free CARS and concurrent SHG and two-photon fluorescence microscopy in an epi detection geometry. Rapid and accurate tuning between Raman resonances was achieved by changing the channels and temperatures of OPA and SHG crystals concurrently with tuning of the CW OPA seed wavelength. The corresponding pump-Stokes frequency detuning range was from 2700 to 3200 ${\mathrm{cm}}^{-1}$, thus fully covering the C-H stretching band that is typically used for the imaging of lipids. The epi-detection capability with an OPA-based setup demonstrates the relevance to biological and clinical samples without the need for extensive sample preparation which would be required to create the thin sections normally used with forward detection.

By combining elements of multiphoton techniques in an inexpensive and compact configuration, our work paves the way for the simple implementation of multimodal CARS with concurrent SHG/TPEF imaging capability. Power level, spectral resolution and dwell times are all comparable with previous reports [1], while utilizing a much simpler setup. In principle, the costs of the system could be further reduced and the tuning range increased by switching to a switchable array of seed diodes as these are readily available in the telecoms wavelength range. This stepping stone points to a lower cost future route to translation of nonlinear laser microscopy imaging for biomedical research and imaging based diagnostics in the clinic. The next steps in the translation of the nonlinear source to clinics and hospitals will involve progressing the system into a robust prototype that can operate in a non-specialised environment. Additionally we will need to progress with clinical feasibility studies with such a microscope system based on the developed laser to demonstrate reproducible performance and visualisation ability for improved diagnosis.
